# High-Performance Infrared Detectors Based on Black Phosphorus/Carbon Nanotube Heterojunctions

**DOI:** 10.3390/nano13192700

**Published:** 2023-10-04

**Authors:** Yanming Zhang, Qichao Li, Xiaowo Ye, Long Wang, Zhiyan He, Teng Zhang, Kunchan Wang, Fangyuan Shi, Jingyun Yang, Shenghao Jiang, Xuri Wang, Changxin Chen

**Affiliations:** National Key Laboratory of Advanced Micro and Nano Manufacture Technology, Key Laboratory for Thin Film and Microfabrication of Ministry of Education, School of Electronic Information and Electrical Engineering, Shanghai Jiao Tong University, Shanghai 200240, China

**Keywords:** photovoltaic devices, diodes, black phosphorus (BP), single-walled carbon nanotubes (SWCNTs), heterojunctions

## Abstract

Infrared detectors have broad application prospects in the fields of detection and communication. Using ideal materials and good device structure is crucial for achieving high-performance infrared detectors. Here, we utilized black phosphorus (BP) and single-walled carbon nanotube (SWCNT) films to construct a vertical van der Waals heterostructure, resulting in high-performance photovoltaic infrared detectors. In the device, a strong built-in electric field was formed in the heterojunction with a favored energy-band matching between the BP and the SWCNT, which caused a good photovoltaic effect. The fabricated devices exhibited a diode-like rectification behavior in the dark, which had a high rectification ratio up to a magnitude of 10^4^ and a low ideal factor of 1.4. Under 1550 nm wavelength illumination, the 2D BP/SWCNT film photodetector demonstrated an open-circuit voltage of 0.34 V, a large external power conversion efficiency (η) of 7.5% and a high specific detectivity (D*) of 3.1 × 10^9^ Jones. This external η was the highest among those for the photovoltaic devices fabricated with the SWCNTs or the heterostructures based on 2D materials and the obtained D* was also higher than those for most of the infrared detectors based on 2D materials or carbon materials. This work showcases the application potential of BP and SWCNTs in the detection field.

## 1. Introduction

Infrared detectors have been widely studied and applied in both military and civilian fields of detection and communication [1,2]. The selection of suitable sensitive materials plays a crucial role in determining the performance of infrared detectors. Black phosphorus (BP) and single-walled carbon nanotubes (SWCNTs) are ideal materials for building infrared detectors. BP has a layered structure with a direct bandgap of 0.3–2.0 eV [3] and exhibits a high mobility of 20,000 cm^2^ V^−1^ s^−1^ [4]. This high mobility can enhance the transition efficiency of the carriers under illumination and the switching speed of the devices [5,6]. The light absorption characteristics of BP shows that BP is a good infrared light-absorbing material [7,8]. Additionally, BP also exhibits good strength, flexibility, and flame retardation [9,10]. Similarly, SWCNTs have a direct tunable bandgap that varies with their diameter [11,12], showing strong photo-absorption range from ultraviolet to infrared [12,13] and a fast light response capability [14]. It has a high mobility up to 100,000 cm^2^ V^−1^ s^−1^ and a conductivity of 10^6^ S m^−1^ [11,15,16]. SWCNTs also exhibit excellent strength and flexibility [11,16].

Some photoconductive infrared detectors based on SWCNTs or BP have been investigated [17,18]. However, a bias needed to be applied in the device for photodetection [19]. A BP/graphene heterostructure had also been used to construct a photovoltaic detector [20]. However, the device sensitivity is relatively low because the monolayer graphene used had a weak photoabsorption [21]. In contrast, a dense SWCNT film has a stronger photo absorption than a monolayer graphene while maintaining a similar carrier mobility as the graphene. SWCNT films and BP also have a good energy-band matching [22]. If BP and SWCNTs can be used together to construct the detectors, it can fully utilize their advantages for achieving high device performance. 

Here, BP and SWCNT films were used to construct vertical van der Waals heterojunctions, resulting in high-performance photovoltaic infrared detectors. In the dark, the devices exhibited a diode-like rectification behavior with a rectification ratio up to a magnitude of 104. Under a 1550 nm wavelength illumination, the 2D BP/SWCNT film device exhibited an open-circuit voltage (V_oc_) of 0.34 V, a high external power conversion efficiency (η) of 7.5%, and a large specific detectivity (D*) of 3.1 × 10^9^ Jones. The obtained external η was the highest among those for the photovoltaic devices fabricated with SWCNTs or the heterostructures based on 2D materials and the D* was also higher than those for most of the infrared detectors based on 2D materials or carbon materials. 

## 2. Materials and Methods

### 2.1. Preparation of the Bulk BP

A short-way transport reaction was used to prepare the bulk BP crystals [23]. First, 20 mg of Sn, 10 mg of SnI_4_ and 0.5 g of red phosphorus were sealed in a quartz ampoule at 0.1 Pa pressure, whose inner diameter was 8 mm. The red phosphorus was the source of the phosphorus and the Sn and SnI_4_ were mineralizers for preparing the BP. The ampoule with the mixed raw materials was put horizontally in a muffle furnace. One end of the mixture sample was placed close to the furnace wall that contained the heating resistance wire, while the other end was near the middle of the muffle furnace. Next, the furnace was heated up to 650 °C and held at 650 °C for 30 min. The furnace was then rapidly cooled down by 10 °C within a few minutes and maintained at 640 °C until the total time was 30 min. In a similar way, we cooled the furnace at a step length of 10 °C until the furnace temperature reached 450 °C. Finally, the furnace was cooled down to room temperature naturally without a heat source.

### 2.2. Fabrication of the 2D BP

The 2D BP sheets were prepared via a mechanical exfoliation method. Firstly, a selected bulk BP crystal was placed on blue tape and exfoliated 7–8 times to obtain thinner 2D BP sheets. During the exfoliation, two pieces of tape were pressed together with finger pressure for 10 s at room temperature and then the tapes were torn off. This was repeated 7–8 times in different areas of the tape. Next, a tape with 2D BP sheets was pressed onto a PDMS substrate (5 mm × 5 mm) by finger pressure at room temperature for 60 s and was then separated from the substrate to obtain 2D BP sheets on the PDMS substrates. Finally, the 2D BP sheets were transferred to a Si substrate by pressing the PDMS with the 2D BP sheets onto the Si substrate and then separating them. The prepared samples were stored in a nitrogen-filled glovebox to prevent the oxidation of the 2D BP until later usage. 

### 2.3. Preparation of the SWCNT Films 

SWCNTs bought from Carbon Solutions Inc. (Riverside, CA, USA) were used in the experiment, which had an average diameter of 1.4 nm (Product Model: P3). Firstly, different weights of SWCNTs were dispersed in 150 mL isopropyl alcohol (IPA) solvent to form a dispersed SWCNT solution by bath sonication at 240 W for 2 h. The SWCNT dispersions were filtered to obtain uniformed SWCNT films. A polycarbonate (PC) filter membrane with a pore size of 0.2 µm was used for the filtration. Then, the SWCNT films with a thickness from dozens of nanometers to hundreds of nanometers were acquired depending on the different concentrations of SWCNT solutions. The PC filter membrane with a SWCNT film was split into 1 mm × 2 mm pieces and were floated on the surface of an N-methylpyrrolidone (NMP) solution for 10 min with SWCNT films on the PC filter membrane. Thus, the PC membrane would react with the NMP solution and be removed, leaving a clean SWCNT film on the surface of the NMP solution. Afterwards, the resulting SWCNT film was transferred into IPA solvent for cleaning, resulting in a clean film on the IPA surface.

### 2.4. Fabrication of the Bulk BP/SWCNT Film Schottky Diodes

First, a bulk BP was placed on a heavily p-doped Si substrate with 100 nm thick thermally oxidized SiO_2_ with dimensions of 1 cm × 1 cm. A drop of IPA was dropped on the BP to wet the interface between the BP and Si and then the sample was kept in IPA at 60 °C for 5 min to fix the BP on the substrate. The SWCNT film was prepared on a PC filter membrane by vacuum filtration. The SWCNT film was separated and floated on the surface of the NMP solvent after dissolving the PC filter membrane. Then, the SWCNT film was aligned on the bulk BP to form the bulk BP/SWCNT film heterostructure via the wet transfer method. The sample was rinsed with IPA and kept for 5 min at 60 °C to dry the film. After that, the positive Au/Cr electrode was fabricated on the bulk BP and the negative Au/Cr electrode was fabricated on the SWCNT film. A thin gold film was deposited on the bottom of the Si substrate to act as the gate. Since instability is a key issue for BP, the devices were stored in a vacuum.

### 2.5. Fabrication of the 2D BP/SWCNT Film Photodetectors

The BP crystals were exfoliated to prepare the 2D BP via mechanical exfoliation on the PDMS. The SWCNT film was fabricated using vacuum filtration. It was transferred to the SiO_2_/Si substrate by the wet transfer method. Next, the 2D BP was then aligned and transferred onto the substrate with a SWCNT film via a dry transfer method. Thus, a 2D BP/SWCNT film van der Waals heterostructure was formed. Finally, the Au/Cr (20 nm thick/3 nm thick) positive and negative electrodes were fabricated on the BP and SWCNT film, respectively, using electron beam lithography (EBL) and the lift-off technique. A thin gold film was deposited on the bottom of the Si substrate to act as the gate. The devices were also stored in a vacuum.

### 2.6. Measurements on the Electrical and Photovoltaic Characteristics of the Devices

The electrical characteristics were tested using an Agilent B1500A (Keysight Inc., Santa Clara, CA, USA) semiconductor performance analyzer at room temperature with a vacuum degree of 8 × 10^−5^ Pa. The devices were tested in dark and illuminated conditions. To obtain the measurements, V_b_ was applied to the BP and the SWCNT film was grounded. To test the photovoltaic characteristics of the device, a 1550 nm wavelength laser (photon energy = 0.80 eV) via a fiber with 8 µm internal diameter was used to illuminate the sensitive area of the devices. The illumination direction was 45° relative to the substrate and the vertical distance from the fiber ends to the substrate surface was 6 mm. The laser output angle was 5°. The laser intensity on the device surface was 6.18 W/cm^2.^ In the experiment, we used a 633 nm laser to identify and guide the position of the light spot.

## 3. Results and Discussion

**Figure 1:**
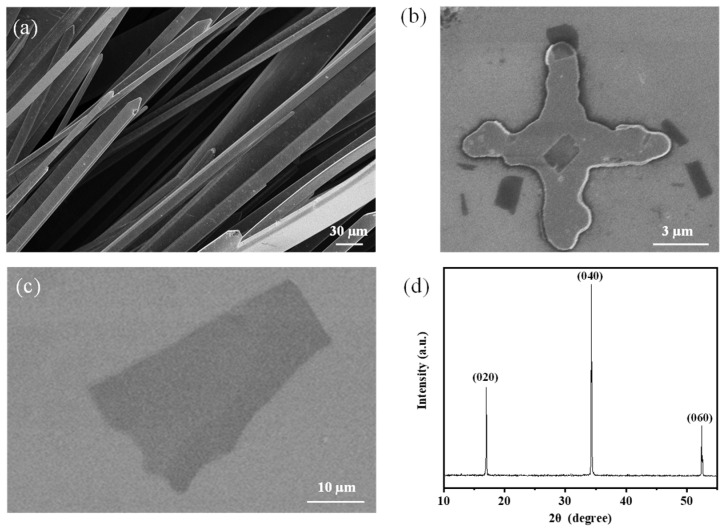
Images and XRD spectrum of the bulk BP crystals. (**a**) SEM image of the BP bunches. (**b**) SEM image of several 2D BP sheets. (**c**) SEM image of obtained 2D BP sheet. (**d**) XRD spectrum of a bulk BP crystal.

The bulk BP crystals were synthetized via a short-way transport reaction mentioned above. The prepared synthetic bulk BP had a metallic luster, smooth surface, and bunch-like shape. The BP bunches consisted of BP crystal branches (Figure 1a). The width of the BP branches was 50 µm to 9 mm, the length of them was 3 mm to 20 mm, and the thickness was a few micrometers to tens of micrometers. The resulting bulk BP crystals were further exfoliated into 2D BP sheets via the mechanical exfoliation method [24]. The prepared 2D BP sheets on the surface of silicon wafers are shown in Figure 1b,c. The cross in Figure 1b is an Au marker. A number of 2D BP sheets could be found in the product. The average area was 1.5 µm^2^. The X-ray diffraction (XRD) spectrum analysis of a bulk BP displayed three sharp BP characteristic peaks of (020), (040), and (060), which indicated the high quality of the grown BP crystals [23].

**Figure 2:**
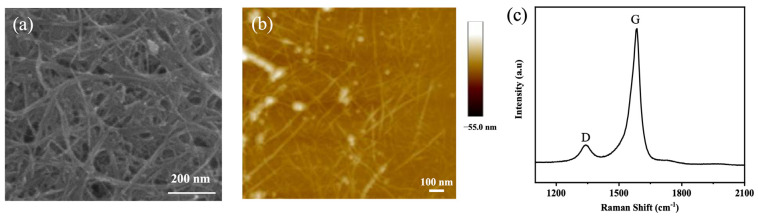
Morphology and Raman spectrum of an obtained SWCNT film. (**a**) SEM image of the SWCNT film on a SiO_2_/Si substrate. (**b**) Zoomed-in AFM image of the SWCNT film. (**c**) Raman spectrum of the SWCNT film.

The SWCNT films were prepared by filtering the SWCNT dispersions. PC filter membranes were used in filtration. The density, thickness, and area of the SWCNT films could be controlled by the concentration of the SWCNT dispersion. The film was then split into 1 mm × 2 mm squares and transferred wet to a SiO_2_/Si substrate (see Experimental Methods). Thus, a film with a dense SWCNT network was obtained (Figure 2b). In the Raman spectrum, the D band and G band of the SWCNTs were located at 1360 cm^−1^ and 1593 cm^−1^, respectively (Figure 2c). It was observed that the SWCNT films exhibit a large G/D intensity ratio, which indicates that there are few defects in the SWCNTs. The sharp G band indicates a high graphitization degree for the SWCNTs.

**Figure 3:**
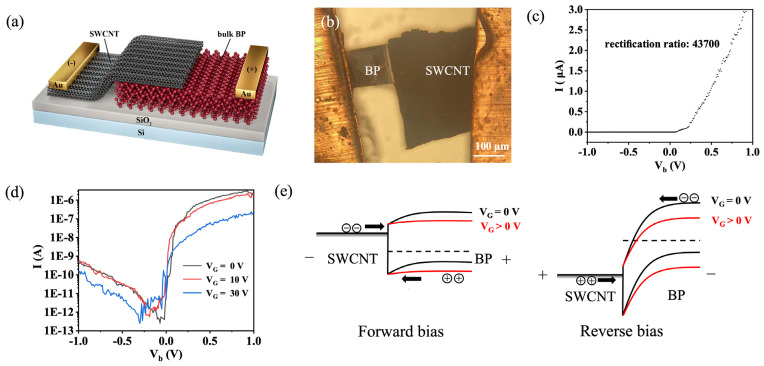
Characteristics and energy-band diagram of the bulk BP/SWCNT film Schottky diode. (**a**) Structural schematic of the device. (**b**) Optical image of the device. (**c**) I–V characteristic curve of the device at V_G_ = 0 V. (**d**) I–V characteristic curve of the diode in different gate voltage. (**e**) Energy band diagrams of the diode under the forward bias (left panel) and the reverse bias (right panel) using different gate voltages.

The bulk BP and SWCNT film were used to form the heterostructure for fabricating the diode (Figure 3a). Figure 3b is an image of the bulk BP/SWCNT film heterojunction diode. A high-quality heterostructure with the SWCNT film aligned on the bulk BP is observed in Figure 3b. The fabricated bulk BP/SWCNT film Schottky diode exhibited good rectification characteristics and gate tunable capability. It was observed that the current increased rapidly under forward bias and a small reverse saturation current of 20 pA was obtained under reverse bias (Figure 3c). A high rectification ratio of 43,700 was obtained when the device bias (V_b_) was ±0.5 V. The following diode formula was used to fit the I–V curve [15],

(1) $$I=I_{0}\exp\left[\frac{q\left(V-{\mathrm{IR}}_{S}\right)}{\mathrm{nkT}}\right]-I_{0}$$

where I_0_ is the reverse saturation current, q is the unit electron charge, R_s_ is the series resistance, n is the ideal factor, k is the Boltzmann constant, and T is the temperature. Thus, a n value of 1.4 and a R_s_ value of 210 kΩ were obtained. The n value for our diode was close to the value of 1 for ideal diodes; small n values can be attributed to the fewer defects and recombination centers in the BP/SWCNT heterojunction. Previous studies have shown that selecting appropriate metals (such as Ni and NiCr) can reduce the contact resistance between metals and BP [25,26]. According to formula 1, the rectification ratio of the diode will increase as the contact resistance decreases. The rectification ratio of the devices is expected to be further improved by selecting the appropriate contact metals.

We also observed that the device had a good gate tunable capability. Both the forward and reverse current decreased as the gate voltage (V_G_) increased from 10 V to 30 V. The rectification ratio also decreased to 20,407 and 1036 at V_G_ = 10 V and V_G_ = 30 V, respectively (Figure 3d). 

The rectification characteristics and the current change under V_G_ for the device were explained as follows. The BP is a p-type semiconductor [27]; the holes acted as the majority carriers and minority carriers (electron) and had very little impact on the results. When the BP contacts the SWCNT, the BP could form a band bend as shown in Figure 3e. When forward bias was added to the diode (Figure 3e, left panel), the holes were transported from the BP to the SWCNT with a small barrier, which caused a large forward current from the BP to the SWCNT. Under reverse bias (Figure 3e, right panel), the hole current was small because there is a large Schottky barrier for holes from the SWCNT to the BP, which caused a small reverse current close to 0 A. Above all, it resulted in a rectification characteristic. The current of the diode was dominated by the hole current since the holes were the majority carriers in the BP. Under forward bias, the hole concentration of the BP decreased as the applied V_G_ increased, which caused the hole current from the BP to the SWCNT to decrease (Figure 3e, left panel). Under reverse bias, as the applied V_G_ increased, the thickness of the BP/SWCNT Schottky barrier increased, which resulted in the hole current from the SWCNT to the BP decreasing (Figure 3e, right panel).

**Figure 4:**
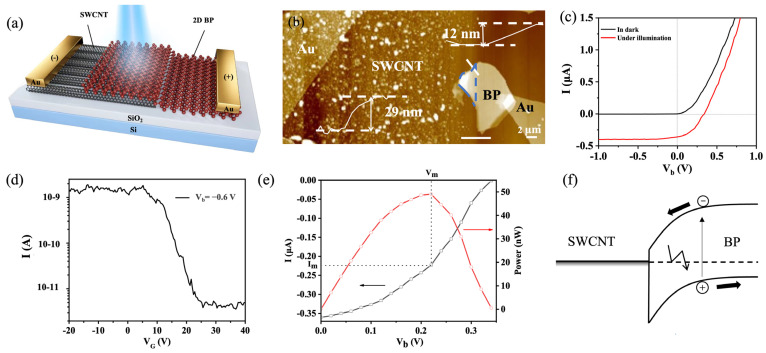
Characteristics and energy-band diagram of the 2D BP/SWCNT film photodetector. (**a**) Structural schematic of the photodetector. (**b**) AFM image of the photodetector. Inset measuring lines: height profile of the SWCNT film and 2D BP was measured along the white line. The overlap area of the device is highlighted by the blue curve. (**c**) I–V curves of the device measured in dark and under illumination at V_G_ = 0 V. (**d**) The device current at V_b_ = −0.6 V as a function of the V_G_. (**e**) The fourth quadrant of the I–V curve and corresponding power generation at under 1550 nm wavelength illumination. (**f**) The energy-band diagram of the BP/SWCNT heterojunction.

A 2D BP with a few layers and a larger band gap were used to construct a 2D BP/SWCNT film heterojunction detector (Figure 4a). The 2D BP acted as the positive electrode and the SWCNT was the negative electrode. Figure 4b shows an atomic force microscopy (AFM) image of the photodetector. The thickness of the 2D BP was measured to be 12 nm and the SWCNT film was about 29 nm. The overlapping area of the 2D BP and the SWCNT film was the sensitive layer, whose area was about 10 µm^2^. A 12 nm BP sheet was estimated to have a bandgap of 0.42 eV according to Ref. [28] and Ref. [29]. The device exhibited a diode-like characteristics in dark conditions (Figure 4c). The rectification ratio of the diode was ~10^3^ at V_b_ = ±1 V and V_G_ = 0 V. We also measured the device current at V_b_ = −0.6 V as a function of the V_G_ (Figure 4d). It was shown that the device had a good gate-tuning capability. At V_b_ = −0.6 V, the current increased as the V_G_ decreased from 40 V to −20 V. A high I_on_/I_off_ ratio of 517 could be obtained for the device.

Under 1550 nm wavelength laser (photon energy = 0.80 eV) illumination, the photodetector exhibited a good photovoltaic effect. A photogenerated current was produced when the device was illuminated by light, whose direction was opposite to that of the forward current (Figure 4c). As the forward bias was equivalent to the V_oc_, the value of the forward current was equal to the absolute value of the photogenerated current, which resulted in a total current of 0 A. The illumination power density of the laser was about 6.18 W/cm^2^, and the photogenerated short-circuit current (I_sc_) and V_oc_ were 0.35 µA and 0.34 V, respectively. The photodetector exhibited a maximum generated power P_max_ = I_m_ × V_m_ = 48 nW, where I_m_ and V_m_ are the output current and voltage at maximum power generation, respectively (Figure 4e).

A figure of merit of the device is the external η, which was calculated by using the following equation [30]:

(2) $$\eta =\left(I_{m}\times V_{m}\right)/P_{\mathrm{in}}=\left(\mathrm{FF}\times I_{\mathrm{sc}}\times V_{\mathrm{oc}}\right)/P_{\mathrm{in}}$$

where P_in_ is the incident power and FF is the fill factor, which indicates the power delivery capability of the photovoltaic device. According to this equation, the FF of the 2D BP/SWCNT film Schottky junction photodetector was about 0.41 under illumination. Within the sensitive area (BP/SWCNT stacking area; Figure 4b blue box), P_in_ was calculated to be 672 nW. The external η of the device was calculated to be 7.5%, which was the highest among the photovoltaic devices fabricated with the SWCNTs or the heterostructures based on the 2D materials [13,21,30,31,32,33]. The detail comparison is shown in Table 1. The physics of the detector could be interpreted by the energy band diagram of the device (Figure 4f). Under illumination, the photogenerated electron–hole pairs in the device were efficiently separated by the strong built-in electric field, resulting in a photogenerated current and voltage and thereby a good photovoltaic effect. 

R and D* represents the sensitivity of the detectors. R represents the ability to convert optical power into electrical signals and the photoresponsivity (R = I_sc_/P_in_) of the photodetector was calculated to be 0.52 A/W. D* describes the ability of the photodetector material to detect the light. D* was calculated using the following Formula [13]:

(3) $$D^{*}=R\sqrt{A}/i_{n}$$

where A is the sensitive area of the photodetector and i_n_ is the root-mean square current noise per bandwidth. in is determined by thermal noise, shot noise from the dark current, and flicker noise [24]. For CNT-based and BP-based photodetectors, the principal factor at zero bias is the thermal noise. Thermal noise could be estimated as [13]:

(4) $$i_{n}=\sqrt{4\mathrm{kT}/R_{D}}$$

where k is the Boltzmann constant, T is the temperature, and R_D_ is the resistance of the diode at zero bias in the dark. According to the curve, R_D_ was calculated to be 530 kΩ; thus, it was 5.6 × 10^−14^ AHz^1/2^/W. Thus, the D* of the detector was calculated to be 3.1 × 10^9^ Jones. The D* value for our device is higher than those for most of infrared detectors based on 2D materials or carbon materials reported in the literature [17,34,35,36]. The summarized D* for our device and the devices in previous reports can be seen in Table 1.

The high external η and D* of the device were attributed to a strong built-in electric field caused in the heterojunction with a favored energy-band matching for the BP and the SWCNTs. The strong light absorption and high mobility of the materials can also enhance the η and D*.

## 4. Conclusions

High-performance diodes and photovoltaic infrared detectors based on a BP/SWCNT film heterojunction were investigated. The fabricated devices exhibited a rectification behavior, whose rectification ratios increased up to a magnitude of 10^4^. Under 1550 nm wavelength illumination, the 2D BP/SWCNT film heterojunction device exhibited a V_oc_ of 0.34 V and an R of 0.52 A/W. The external η and D* of the device were 7.5% and 3.1 × 10^9^ Jones, respectively. The obtained external η was the highest among those for the photovoltaic devices fabricated with SWCNTs or heterostructures based on 2D materials. The D* value was also higher than those for most of the infrared detectors based on 2D materials or carbon materials. This work demonstrates that BP and SWCNTs have vast application potential in the photodetection field.

## Figures and Tables

**Table 1:** Comparison of the device in this work and the devices fabricated with SWCNTs or 2D materials in previous reports.

Photosensitive Material of the Device	D*/Jones	External η/%	Incident Wavelength/nm	Device Type	Source
2D BP/SWCNT film heterostructure	3.1 × 10^9^	7.5	1550	Photovoltaic	This work
BP nanoflake	1.83 × 10^8^	/	830	Photoconductive	17
carbon nanotube (CNT) parallel array	1.09 × 10^7^	0.0156	785	Photovoltaic	18
CNT thin film/graphene Schottky junction	1.0 × 10^8^	/	980	Photovoltaic	34
graphene/multiwall CNT nanohybrid	1.5 × 10^7^	/	1300	Photovoltaic	35
2D tellurium (Te)	2.0 × 10^9^	/	1700	Photoconductive	36
BP/MoS_2_ heterosturcture	/	0.195	830	Photovoltaic	32
Pd/CNT/Al junction	/	5.0 (intrinsic η)	1550	Photovoltaic	31
Pd/CNT/Sc junction	/	0.11	785	Photovoltaic	13

## Data Availability

The data presented in this study are available on request from the corresponding author.
